# Moderate Nucleoporin 133 deficiency leads to glomerular damage in zebrafish

**DOI:** 10.1038/s41598-019-41202-4

**Published:** 2019-03-20

**Authors:** Chiara Cianciolo Cosentino, Alessandro Berto, Stéphane Pelletier, Michelle Hari, Johannes Loffing, Stephan C. F. Neuhauss, Valérie Doye

**Affiliations:** 1 Institute of Molecular Life Sciences, University of Zurich, Zurich, Switzerland; 2 Institut Jacques Monod, UMR7592 CNRS-Université Paris Diderot, Sorbonne Paris Cité, F-75205 Paris, France; 3 Ecole Doctorale SDSV, Université Paris Sud, F-91405 Orsay, France; 4 Institute of Anatomy, University of Zurich, Zurich, Switzerland; 5 Fondazione RiMED, Palermo, Italy

**Keywords:** Nuclear pore complex, Organogenesis

## Abstract

Although structural nuclear pore proteins (nucleoporins) are seemingly required in every cell type to assemble a functional nuclear transport machinery, mutations or deregulation of a subset of them have been associated with specific human hereditary diseases. In particular, previous genetic studies of patients with nephrotic syndrome identified mutations in *Nup107* that impaired the expression or the localization of its direct partner at nuclear pores, Nup133. In the present study, we characterized the zebrafish *nup133* orthologous gene and its expression pattern during larval development. Using a morpholino-mediated gene knockdown, we show that partial depletion of Nup133 in zebrafish larvae leads to the formation of kidney cysts, a phenotype that can be rescued by co-injection of wild type mRNA. Analysis of different markers for tubular and glomerular development shows that the overall kidney development is not affected by *nup133* knockdown. Likewise, no gross defect in nuclear pore complex assembly was observed in these *nup133* morphants. On the other hand, *nup133* downregulation results in proteinuria and moderate foot process effacement, mimicking some of the abnormalities typically featured by patients with nephrotic syndrome. These data indicate that *nup133* is a new gene required for proper glomerular structure and function in zebrafish.

## Introduction

Efficient and regulated bidirectional transport between the cytoplasm and the nucleus is an essential process in all eukaryotic cells. This function is achieved by nuclear pore complexes (NPCs), huge assemblies anchored within the nuclear envelope and composed of about 30 different proteins, termed nucleoporins (Nups) (reviewed in^1^). Despite the universal role of NPCs in all nucleated cells, some Nups are linked to human hereditary diseases affecting specific cell types or organs (reviewed in^2–4^).

In particular, genetic studies have implicated a restricted number of structural nucleoporins in specific kidney diseases termed nephrotic syndromes (NS). NS arise from defects or damages that impair the selectivity of the glomerular filtration barrier and lead to massive proteinuria and hypoalbuminemia, which in turn cause edema and dyslipidemia. The glomerular filtration barrier (GFB) surrounds the glomerular capillaries and comprises three layers: (i) a fenestrated endothelium, (ii) a basement membrane, and (iii) the podocytes. The latter are highly specialized epithelial cells characterized by long and thin cytoplasmic projections, termed foot processes (FPs), that interdigitate and are connected by specialized cell-cell junctions, the slit diaphragms (reviewed in^5^). While most patients with childhood-onset NS respond well to steroid treatments, 10–20% of the affected children do not achieve remission upon corticosteroid therapy. Steroid-resistant NS (SRNS) is associated with a high risk of progression to end-stage renal disease (ESRD)^6^. It frequently manifests histologically as focal segmental glomerulosclerosis (FSGS), characterized by scattered scarring of some glomeruli and is often associated with retractions (“effacement”) of podocytes foot processes (reviewed in^7^).

Although nonhereditary forms of SRNS seem to be prevalent, studies over the last years have identified over 50 dominant or recessive single-gene mutations in a significant percentage (30%) of patients with early-onset SRNS and FSGS (reviewed or discussed in^6,8–11^). While some of these genes have podocyte-specific or -restricted functions, these studies also unveiled the implication of multiple cellular processes in the establishment or maintenance of the glomerular filtration barrier^7,12,13^. In particular, these genetic studies have identified mutations in Nup93 and Nup205, two constituents of the inner ring of the NPC^14^ and in Nup107, a constituent of the Y-complex (Nup107/160-complex) that builds up the cytoplasmic and nuclear rings of the NPC^15–17^. Mutations within *Nup107* were also identified in patients with a rare co-occurrence of microcephaly with nephrotic syndrome, similar to Galloway-Mowat syndrome (GAMOS)^18^. While patients with GAMOS-like presentation had a strong reduction in Nup107 protein level accompanied by decreased levels of Nup133, its direct partners within the Y-complex^18^, another SRNS-linked mutation affecting Nup107 was shown to impair its interaction with Nup133^16^. These data thus pointed towards a possible implication of Nup133 in NS.

In mice, a previous characterization of a *Nup133* null mutant (*mermaid, or merm*) revealed that mouse embryos lacking a functional *Nup133* allele developed through midgestation but die at e9.5–e10.5^19^. While this indicates that Nup133 is not an obligate NPC component, *merm* mutants displayed abnormalities in a number of tissues, indicating that cell differentiation towards several epiblast-derived lineages likely requires Nup133^19^. However, the possible contribution of Nup133 to kidney development or function has never been assessed. To address this question, we used morpholino-mediated *nup133* inactivation in zebrafish (*Danio rerio, Dr*), a well-established vertebrate model to study kidney development and renal diseases^20–23^. We report here that limited knockdown of zebrafish *nup133*, while not impairing early stages of kidney development, leads to glomerular abnormality that mimic nephrotic syndrome.

## Results

### Zebrafish *nup133* ortholog is broadly expressed at early stages and becomes restricted to specific tissues at later stages

Query of the latest version of the genome databases identified a unique *nup133* orthologue gene in zebrafish, *Dr nup133* (ZFIN:ZDB-GENE-040426-2941; Ensembl:ENSDARG00000010078) containing 26 exons located on the forward strand of chromosome 1. The open reading frame is predicted to encode a protein of 1136 amino acids (aa) that shares 62.3% overall amino acid identity with human Nup133 (Supplementary Fig. S1).

**Figure 1:**
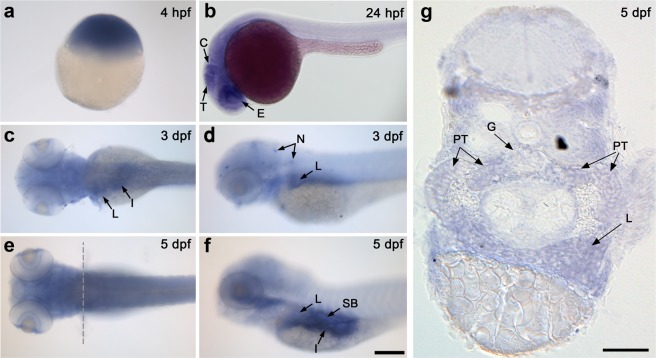
Expression of *nup133* in the developing zebrafish detected by *in situ* hybridization (ISH). Whole mount ISH with *nup133* antisense probe of embryos at: (**a**) sphere stage (4 hpf; embryo shown with animal pole to the top); (**b**) 24 hfp (lateral view); (**c**–**f**) 3 and 5 dpf (left panels: dorsal view; right panels: lateral view). Arrows point to tissues with enriched expression of *nup133*. Abbreviations: E: eyes; T: tectum; C: cerebellum; L: liver; I: intestine; N: neuromasts; SB: swim bladder. Scale bars, 200 µm. (**g**) Transverse section of a 5 dpf embryo at the level of the pectoral fins (as shown in the dotted line in **e**) confirms *nup133* expression in the liver, and show in addition a diffuse staining in the proximal tubules (PT) and a faint signal in the glomerulus (G). Scale bar, 50 µm.

To determine the spatio-temporal localization of *nup133* transcripts in zebrafish embryonic tissues, we performed whole-mount *in situ* hybridization (ISH) at different developmental stages (Fig. 1a–f). The *nup133* sense RNA probe was used as negative control (Supplementary Fig. S2). Ubiquitous expression of *nup133* was observed at sphere stage (4 hours post fertilization, hpf, Fig. 1a and Supplementary Fig. S2). By 24 hpf, *nup133* mRNA was detected in the central nervous system, with higher levels of expression in the retina, the tectum and the cerebellum (Fig. 1b). At 3 days post fertilization (dpf), in addition to a diffuse staining notably in the brain, we found evidence of *nup133* mRNA enrichment in the liver, in the intestine and in neuromasts of the lateral line organ (Fig. 1c,d). At 5 dpf, the overall expression of *nup133* is weaker, but the mRNA is still detectable in the brain, and enriched in the liver, the intestine and the swim bladder (Fig. 1e,f). Cross sections at 4 and 5 dpf confirmed the enrichment of *nup133* mRNA in the liver and revealed the presence of *nup133* mRNA also in the pronephric proximal tubules and the glomerulus, albeit in low amounts (Fig. 1g, and Supplemental Fig. S2). Consistent with these ISH data and with a previous genome wide RNA-seq dataset^24^ quantitative RT-PCR performed at 1, 2 and 5 dpf revealed a progressive decrease of *nup133* mRNA level during development as compared to beta2 actin mRNA (*actb2*, previously reported to be extremely stable at these stages of zebrafish development (Supplementary Fig. S2f)^24,25^.

### *Partial loss of nup133* causes glomerular cysts in zebrafish

**Figure 2:**
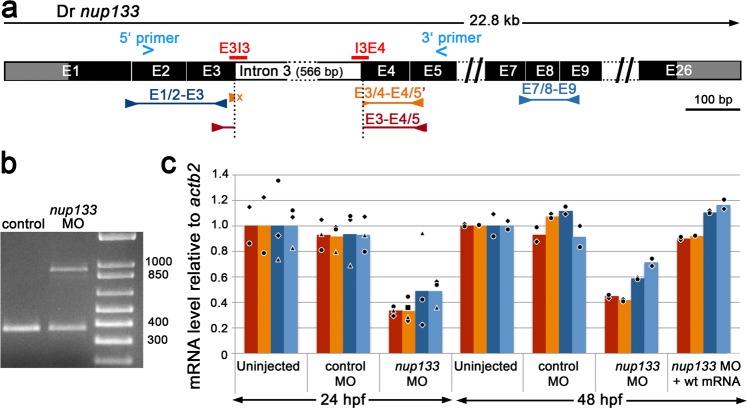
Splice Morpholinos (MO) targeting *nup133* lead to a partial degradation of *nup133* mRNAs. (**a**) Exon structure of *Danio rerio* (*Dr*) *nup133* around the binding sites of the E3I3 and I3E4 splice morpholinos. Blue arrowheads above the scheme indicate the position of the RT-PCR primers used in (**b**) and blue/red/orange arrows below indicate the position of primers and RT-qPCR products used in (**c**). The size of intron 3 is indicated. (**b**) RT-PCR from total RNAs of 48 hpf uninjected embryos (control) and of embryos coinjected with the two splicing morpholinos (*nup133* MO) reveal an additional band caused by retention of intron 3. (**c**) *nup133* mRNA levels relative to *actb2* expression was determined by RT-qPCR on 24 and 48 hpf embryos, uninjected, injected with control MO or *nup133* MO, or sequentially injected with *3xHA-mCherry*-*Dr nup133* mRNA (wt mRNA) and *nup133* MO. The E3-E4/5 (red) and E3/4-E4/5′ (orange) primer pairs only amplify *nup133* mRNA with properly spliced intron 3 (i.e. wt *nup133* mRNA not targeted by the morpholinos) while the E1/2-E3 and E7/8-E9 primer pairs (blue bars) recognize both the spliced and unspliced mRNAs.

In order to characterize the *in vivo* function of *nup133* in zebrafish larvae, we generated knockdown larvae using splice-blocking antisense morpholino oligos (MO) targeting the exon-intron boundary (splice donor E3I3) of exon 3 and the intron-exon boundary of exon 4 (splice acceptor I3E4) of the *nup133* gene (Fig. 2a). Reverse transcription-PCR (RT-PCR) demonstrated that the morpholinos interfere with the splicing of exon 3, as revealed by the sequencing of the additional RT-PCR product detected in MO-treated compared to control embryos (Fig. 2a,b and Supplemental Fig. S3). Retention of intron 3 in *nup133* transcripts is predicted to produce a truncated protein of 124 aa. Quantitative analyses revealed that the levels of properly spliced *nup133* mRNA (red and orange bars in Fig. 2c) drop to about 30% and 40% in 24 and 48 hpf embryos respectively upon *nup133* MO injection as compared to control embryos (uninjected or injected with control MO). These MO-injected embryos only contained 20–30% of intron 3-containing *nup133* mRNAs (difference between the red/oranges and blue bars in Fig. 2c), indicating that most of the intron 3-containing mRNAs, which encode premature stop codons, are degraded via non-sense mediated mRNA decay. Western blot analysis confirmed these data by revealing a clear albeit moderate decrease of Nup133 protein level in 24 hpf embryos (Supplemental Fig. S3).

**Figure 3:**
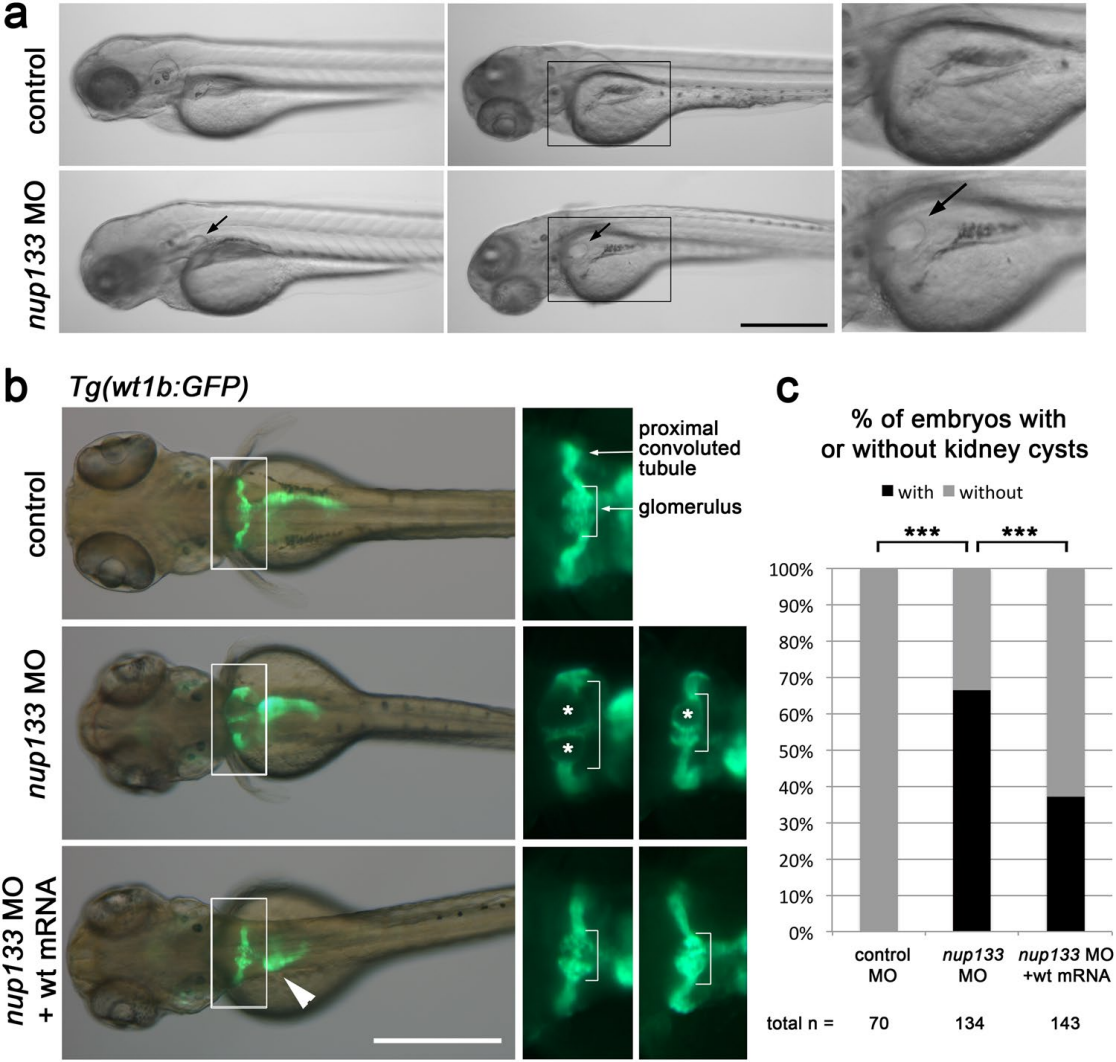
Partial knockdown of *nup133* causes glomerular expansion in zebrafish. (**a**) Gross morphology of 3 dpf control and *nup133* MO embryos (left panels: lateral view; middle panel: dorso-lateral view from two distinct embryos). Scale bar, 500 µm. Two-fold magnification of the indicated area is shown in the rightmost panels. Arrows indicate the pronephric cysts detected in the *nup133* MO embryos. (**b**) Dorsal view of 3dpf *Tg(wt1b:EGFP)* embryos uninjected (control, top panels), injected with *nup133* MO (middle panels), or sequentially injected with *3xHA-mCherry*-*Dr nup133* mRNA (wt mRNA) and *nup133* MO (bottom panels). Overlays of transmission (gray) and GFP-signal (green) images reveal the glomerulus, proximal tubules, and exocrine pancreas. Scale bar, 500 µm. Two-fold magnification of the indicated area and of the same area from a distinct larvae are shown in the right and rightmost panels, respectively. The glomerular structure is indicated in brackets. Asterisks point to cystic dilations of the pronephros in *nup133* MO (middle panels). Note that the *nup133 MO* + wt mRNA embryo displays a left-sided exocrine pancreas (arrowhead) (see also Supplemental Fig. S5). (**c**) Relative proportion of embryos showing or not kidney cysts at 3 dpf. For each condition, the total number of embryos analyzed is indicated (n=, quantified in 2 distinct experiments for control MO injections and 5 experiments for *nup133* MO injections. See also Supplementary Fig. S3). Unlike the embryos injected with control MO, those injected with *nup133* MO frequently feature kidney cysts. On the other hand *nup133* MO + *wt* mRNA showed significantly fewer cysts than *nup133* MO alone. ***P < 0.0001 using a Fisher exact probability test.

Following *nup133* MOs injections, zebrafish larvae frequently developed pericardial edema and exhibited an expansion of the glomerulus detectable at 3 dpf by the formation of pronephric cysts (Fig. 3a and Supplemental Fig. S3). Using the *Tg(wt1b:eGFP)* transgenic line^26^, in which podocytes and proximal pronephric tubules express EGFP under the *wt1b* promoter, the glomerular expansion could be directly observed under a fluorescence microscope (Fig. 3b,  asterisks). Analysis of semi-thin transverse sections of the glomerulus and proximal tubules at 5 dpf confirmed that the Bowman’s space of the glomerulus was dilated in *nup133* morphants compared with the control larvae (Supplemental Fig. S3). To establish the specificity of MOs effects, we determined whether *nup133* MO phenotypes could be rescued by co-injection of a synthetic zebrafish *nup133* (*Dr nup133*) mRNA. The *Dr nup133* mRNA was fused to a triple-hemagglutinin (HA) epitope and mCherry that enabled us to confirm the expression of the resulting 3xHA-mCherry-Dr Nup133 protein by western blot (Supplementary Fig. S4). Co-injection of the splice MOs with the *3xHA-mCherry-Dr nup133* mRNAs reduced significantly the percentage of larvae with glomerular cysts (Fig. 3c and Supplementary Fig. S4). This demonstrates that the glomerular phenotype observed in *nup133* morphants is due to a specific effect of *nup133* knockdown.

In the course of this study, we also noticed that the exocrine pancreas, an organ normally positioned on the right side of the zebrafish embryo and visualized by the *wt1b:GFP* transgene in 3 dpf embryos, was misplaced in about 15–20% of the *nup133* morphants (Supplemental Fig. S5). Analysis of heart looping in 2 dpf embryos confirmed this Left-Right patterning defect^27^ (Supplemental Fig. S5). Importantly however, and unlike the frequency of kidney cysts, these Left-Right patterning defects that are classically linked to ciliopathy were not rescued by injection of the *3xHA-mCherry-Dr nup133* mRNA (Arrowhead in Fig. 3b and Supplemental Fig. S5). This indicates that the appearance of kidney cysts upon partial *nup133* knockdown is not correlated with a Left-Right patterning defect and may therefore not result from a primary defect in cilia assembly or function (see discussion).

Finally, because mutations of Nup107 that affect the stability of Nup133, also leads to microcephaly in human patients^18^, we measured the size of the heads of control, *nup133* MO and *nup133 *MO + wt *nup133* mRNA embryos at 3 dpf. While a very mild decrease of head width and length was observed in *nup133* morphants as compared to control embryos, this defect was not rescued by injection of *nup133* mRNA (Supplemental Fig. S5). This slight delay in development may thus reflect unspecific effects of the morpholinos^28^ rather than a specific effect of *nup133* downregulation.

### *nup133* morphants do not feature major NPC assembly defects and properly express molecular markers of kidney development

**Figure 4:**
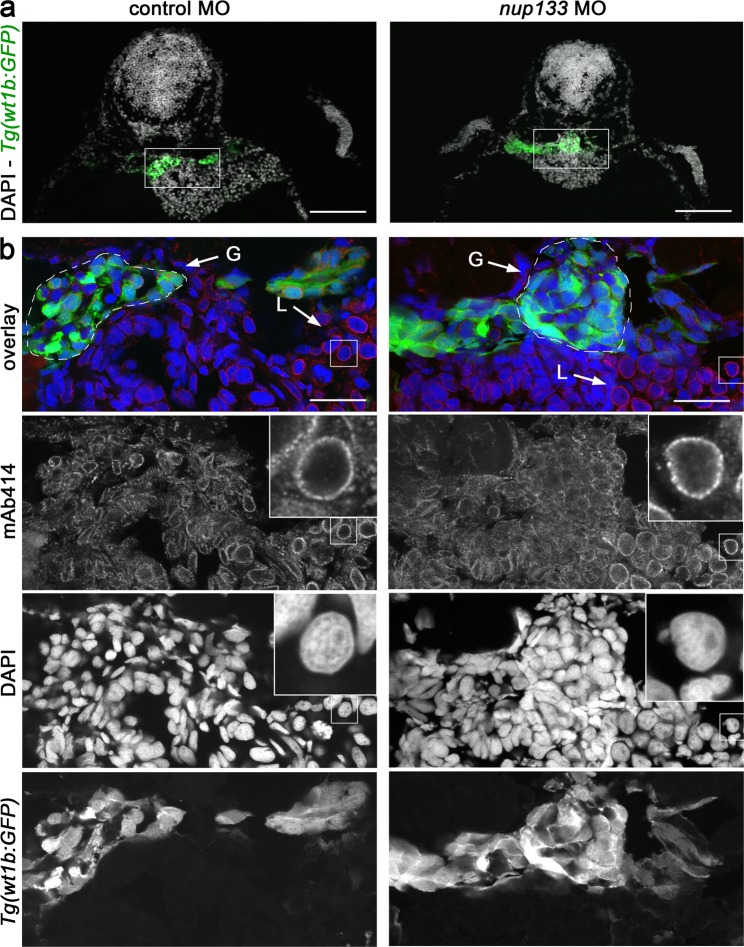
Nup133 depletion does not result in major nuclear pore complex assembly defects. (**a**) Representative transverse sections of the glomerulus of 3 dpf *Tg(wt1b:EGFP)* embryos treated with either control or *nup133* morpholinos (MO) and stained with mAb414 (not shown in these panels) and DAPI. In these sections, GFP-positive cells mark the glomerulus and the neck region of the proximal tubules. Scale bars, 100 μm. (**b**) Fivefold magnification of the areas indicated in (**a**) encompassing the glomerulus (G, indicated by the dashed lines) and the liver (L). mAb414 antibody, that recognizes a subset of FG-nucleoporins, shows specific staining around the nucleus of all cells (visualized with DAPI), with however cell-type-dependent variations in intensity. Note for instance the more prominent staining of liver as compared to glomerular cells. In contrast, no major difference can be seen between control and *nup133* MO-treated embryos. Insets show a fourfold magnification of representative liver nuclei, revealing the punctate staining typical of NPC staining (mAb414). Scale bars, 20 μm.

Because Nup133 is a structural nucleoporin, we next analyzed the consequence of its partial depletion on NPC assembly, using as readout the mAb414 antibody that recognizes a subset of FG-containing nucleoporins^29^. We therefore stained cryosections of 3 dpf *Tg(wt1b:EGFP)* embryos either treated with control MO or treated with *nup133* MO and featuring detectable kidney cysts. While this analysis uncovered a large variability of staining intensity in-between tissues in the control embryos, it did not reveal major alterations of the NPC density in the *nup133* MO compared to control MO-treated embryos (Fig. 4). Although specific alteration of a given nucleoporin cannot be excluded, these data suggest that unlike previously reported for Nup107 depletion in zebrafish^30^ the partial depletion of Nup133 does not affect the overall assembly of NPCs.

**Figure 5:**
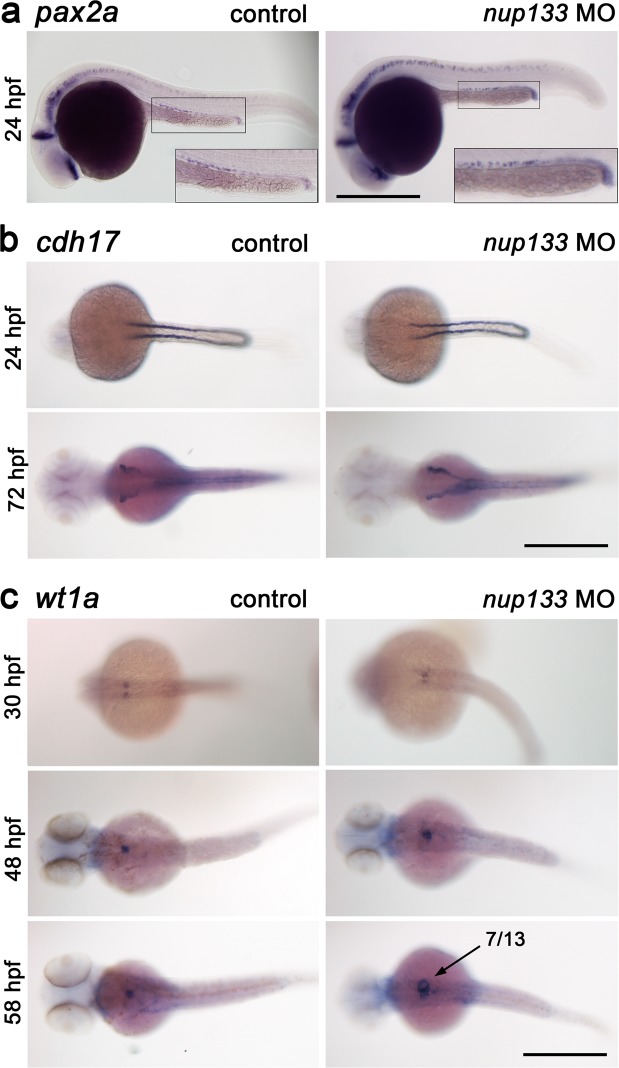
Normal development of the pronephric tubules and glomerulus in *nup133* morphants. (**a**) Lateral view of *pax2a* mRNA expression in pronephric tubules at 24 hpf in uninjected (control) and *nup133* MO-injected embryos reveal that the developmental expression of *pax2a* is not altered in *nup133* morphants. Two-fold magnification of the pronephric tubules is also shown. (**b**) Pronephros marker *cdh17* mRNA expression in *nup133* MO is comparable to that of uninjected controls at 24 and 72 hpf (dorsal view). (**c**) Glomerular development in control and *nup133* MO embryos visualized using the podocyte differentiation marker *wt1a* (dorsal view). At 30 hpf (upper panels), *wt1a* marks future podocytes with two distinct domains in both control and *nup133* MO. At 48 hpf (middle panels), the glomerular primordia merge to the midline to form a single glomerulus. At these stages, mRNA expression does not differ between control and *nup133* MO (middle panels). At 58 hpf (lower panels), after the onset of glomerular filtration, the increased area labeled by the *wt1a* probe (arrow) reflects the glomerular expansion observed in 7 out of 13 *nup133* morphants analyzed. Scale bars, 500 µm.

We next wanted to determine whether the glomerular expansion in *nup133* morphants is caused by a developmental defect of the glomerulus and/or of the pronephros. For this purpose, we used whole-mount ISH to screen established markers of pronephros and glomerulus development. We first assayed expression of the intermediate mesoderm marker *pax2a* (*paired box gene 2a*). At 24 hpf, *pax2a* is expressed during early somitogenesis in the developing pronephric tubules and is important in establishing the boundary between podocytes and the neck segment of the nephron^31,32^. In *nup133* morphants, expression of *pax2a* at 24 hpf was similar to that of wild type embryos, suggesting that the tubular development is not compromised in the morphants (Fig. 5a). We further examined the pronephric tubules and ducts in *nup133* knockdown embryos by checking the expression of the kidney specific marker *cadherin 17* (*cdh17*) at 24 hpf and 72 hpf. Again, no obvious difference in expression of *cdh17* was observed between control and *nup133* morphant larvae at 24 or 72 hpf (Fig. 5b). Based on these markers, we conclude that tubular development is not impaired upon *nup133* knockdown.

We next used an mRNA probe for *Wilms tumor suppressor 1a* (*wt1a*) that is predominantly expressed in podocytes throughout pronephric development^33^. At 24 hpf, *wt1a* is still expressed in two distinct domains, the glomerular primordials, that fuse to form a single glomerulus by 40 hpf^34,35^. In *nup133* morphants, expression of *wt1a* was comparable at 30 and 48 hpf to that of wild type embryos (Fig. 5c). *wt1a* expression sometimes revealed some glomerular enlargement already at 48hpf, a phenotype that became more evident at 58 hpf, after the onset of the glomerular filtration (Fig. 5c, bottom panels). Nevertheless, *wt1a* transcripts were still expressed in *nup133* morphants podocytes. These results suggest that knockdown of *nup133* does not affect the gross development of zebrafish glomerulus.

### *nup133* deficiency affects the normal function of the glomerular filtration barrier

**Figure 6:**
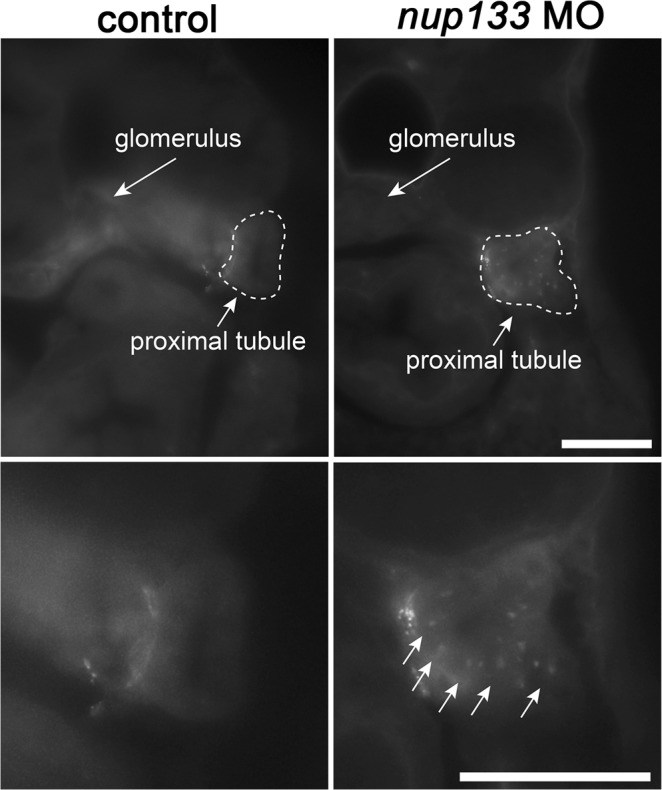
Glomerular filtration is impaired in *nup133* morphants. Representative images of cross sections of 4 dpf control (left panels) and *nup133* morphants larvae (right panels) fixed 20 minutes after injection of Alexa Fluor™ 647 conjugated-BSA into the common cardinal vein. Lower panels represent a higher magnification view of the proximal tubule region (dotted lines). Note the uptake of fluorescent BSA in the apical endosomes of the proximal tubules of the Nup133-depleted larva (arrows). Scale bars 50 µm.

Because the glomerular enlargement upon *nup133* knockdown becomes striking at the onset of glomerular filtration, we next analyzed the functionality of the pronephros in the morphant larvae. The glomerular filtration barrier (GFB) of the kidney allows the free filtration of water and small solutes, while restricting the flow of large plasma proteins such as albumin^36^. However, if the GFB is damaged, albumin and other large proteins pass the barrier to a great extent. Part of the albumin is endocytosed by the epithelial cells of the proximal tubules, while the rest gets then excreted via the final urine leading to proteinuria. In zebrafish larvae, it is possible to inject fluorescent compounds of specific size into the general circulation and then to monitor the appearance of fluorescent endosomes in the apical cytoplasm of pronephric tubule cells^37,38^. To assay for kidney function, we therefore injected fluorescently labeled albumin from Bovine Serum (BSA, Alexa Fluor™ 647 conjugate) into the common cardinal vein of wild type and *nup133* morphant larvae at 4 dpf and then examined the appearance of fluorescent endosomes in the cytoplasm of pronephric tubule cells. Larvae were fixed 20 minutes after BSA injections and sections of the pronephric proximal tubules were imaged. In the control larvae (n = 6, arising from two distinct experiments), we did not observe fluorescent signal from the injected BSA in the apical endosomes of the proximal tubules, indicating that BSA was not able to pass through the intact filtration barrier (Fig. 6, left panels). In most (5/6 imaged larvae) *nup133* morphants, however, fluorescently labeled endosomes were detectable in the proximal tubules (Fig. 6, right panels). This suggests that the filtration barrier of the glomerulus is impaired upon *nup133* knockdown, allowing the passage of macromolecules with a size exceeding the size selectivity of an intact GFB, a condition defined in patients as proteinuria. A statistically relevant analysis of phenotypic rescue would have required BSA injection and imaging of more than 100 embryos and was thus not performed. However, the BSA filtration defects observed in the *nup133* morphants are consistent with the presence of kidney cysts (a phenotype that we could successfully rescue with injections of mRNA). This suggests that the altered functionality of the GFB is most likely a specific consequence of *nup133* knockdown.

### *nup133* knockdown leads to ultrastructural abnormalities of the glomerular filtration barrier

**Figure 7:**
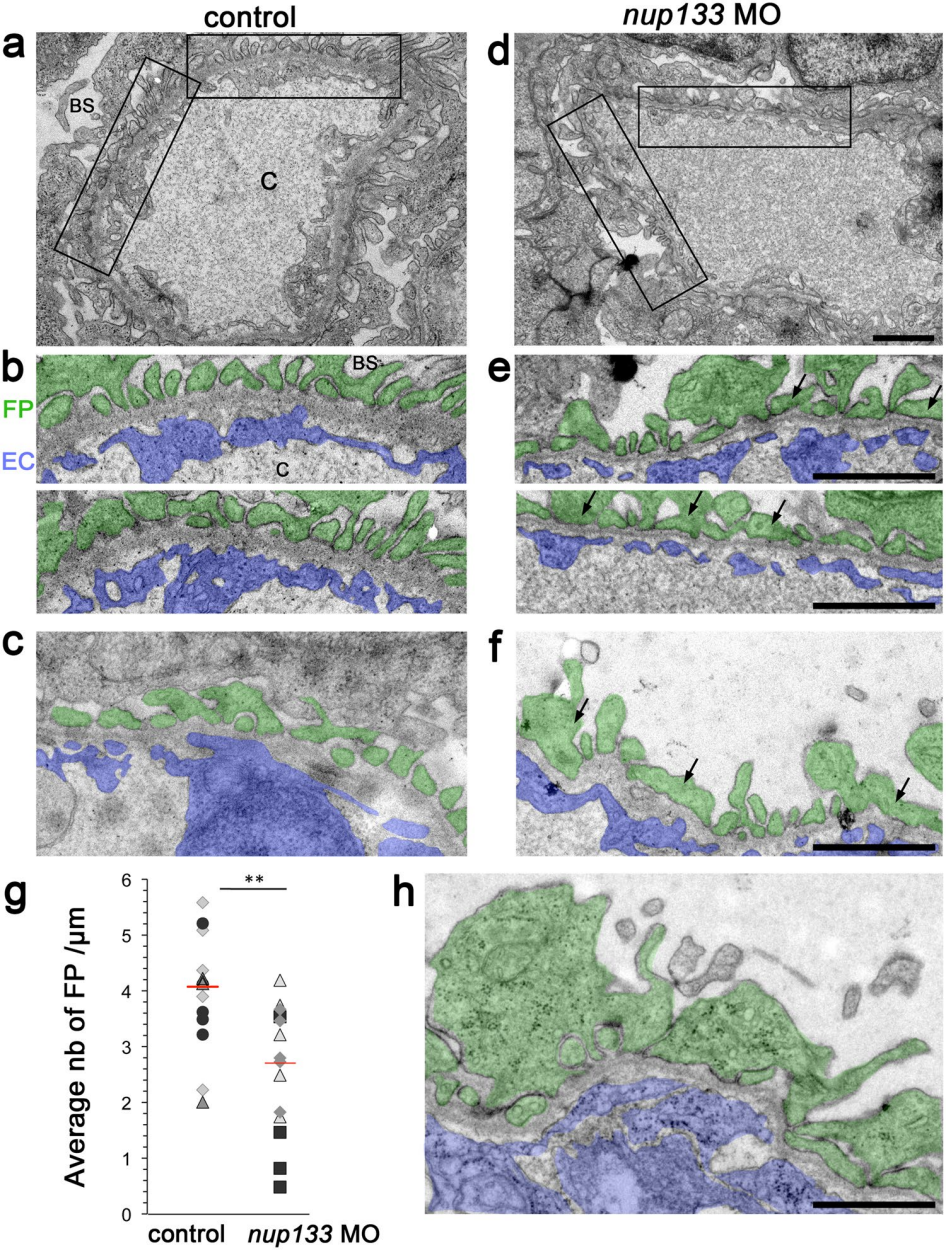
*nup133* knockdown induces moderate foot processes effacement. (**a,d**) Transmission electron micrographs showing the glomerular capillary wall of 5 dpf uninjected control (**a**) and *nup133* MO injected larvae (**d**). (**b,e**) Two-fold magnification of the areas indicated in (**a,d**), and (**c,f,h**) representative images from distinct larvae. Images were pseudocolored to better highlight the glomerular filtration barrier components (podocyte foot processes in green, fenestrated endothelium in blue). Arrows point to irregular-shaped foot processes that are more frequently found in *nup133* MO larvae. (**g**) Quantification of foot processes (FP) density along the basement membrane, presented as average number of FP/µm, was performed as described under materials and methods. The distinct labels reflect values measured on the 3 wt or morphant embryos. Statistical analyses were performed using Wilcoxon-Mann-Whitney Rank Sum Test. **P ≤ 0.01 (p = 0.002486). Image (**h**) corresponds to a basement membrane surrounding a large cyst (presented in Supplemental Fig. S6e). Note that statistical analyses performed without the latter *nup133* morphant larva (values indicated by black squares on the graph) still revealed a significant difference between the control and the *nup133* morphants (p = 0.01855). Abbreviations: BS: bowman space; C: capillary lumen; EC: endothelial cells; FP: foot processes. Scale bars 1 µm.

In order to determine whether the glomerular filtration impairment observed in *nup133* morphants was a consequence of a defect in the glomerular filtration barrier (GFB), we conducted electron microscopy studies to compare the ultrastructure of the glomerulus in *nup133* morphants and wild type larvae at 5 dpf, a time point when the larval glomerulus development is considered nearly complete^34,39^ (Fig. 7 and Supplementary Fig. S6). In wild type larvae, the glomerulus showed the typical organization with podocytes harboring well-developed interdigitated foot processes on the outer side, and fenestrated endothelial cells on the inner side of the glomerular basement membrane (GBM) (Fig. 7a–c). In *nup133* knockdown larvae with kidney cysts, however, irregularly-shaped processes covering the GMB were frequently observed, although typical foot processes were still present in some regions (Fig. 7d–f). In addition to this moderate foot process effacement, more extreme alterations were observed around the expanded bowman space (Fig. 7h, and Supplementary Fig. S6). In the latter case, podocyte cell bodies in direct contact with the GBM were frequently observed (Supplementary Fig. S6). Quantification of foot processes density along the basement membrane confirmed the disorganization of podocyte foot process architecture in the *nup133* morphants (Fig. 7g). Observation of proximal tubules (PTs) revealed variable phenotypes in the morphants: a normal organization, with well-organized microvilli and ciliated cells was observed in morphants with a mild phenotype (Supplementary Fig. S7); in contrast larvae presenting a more severe phenotype featured also an expanded lumen and a less organized brush border (Supplementary Fig. S7). Multiple cross sections of cilia were nevertheless observed inside these expanded PT lumens (Supplementary Fig. S7). These ultrastructural data, combined with the observation of leakage and recapture of fluorescently labeled albumin, suggest that zebrafish *nup133* is mainly required for the structural integrity of the GFB and that the observed defects in proximal tubules are secondary to an altered GFB.

## Discussion

In this study we used zebrafish as model organism to evaluate the contribution of Nup133 to vertebrate kidney function. Using splice-blocking MO-based depletion of Nup133 followed by rescue experiments with *nup133* mRNA, we have identified *nup133* as a new regulator of glomerular structure and functional integrity.

Our study of glomerulus and proximal tubules expression markers shows that the decreased level of Nup133 does not affect the initial stages of nephron development. In addition, TEM analysis of the glomerulus of Nup133-depleted zebrafish reveals that all the cell types constituting the GFB, including the podocytes, are in place. However, the podocytes of Nup133-depleted zebrafish present some degrees of effacement that correlate with a defective GFB. This result therefore suggests that Nup133 plays a fundamental role in the maintenance rather than in the establishment of the GFB.

While we describe here a kidney-specific alteration caused by a moderate depletion of Nup133, Fujita *et al*.^40^ very recently reported a translation-blocking morpholino leading to a more efficient depletion of Nup133 protein level in zebrafish and causing both glomerular defects and microcephaly. On the same line, more severe developmental phenotypes were previously observed upon *Nup133* inactivation in mouse^19^. Similarly, while MO-induced depletion of zebrafish Nup107 mainly causes glomerular defects^16^, transposon insertion in *nup107*^*tsu068Gt*^ transgenic embryos affects multiple tissues including the pharyngeal skeleton, the intestine, the swim bladder and the eyes^30,41^, and a CRISPR/Cas9-generated homozygous truncating “null” mutation of *nup107* showed early lethality at 5 dpf associated with developmental malformations^42^. Together, these data indicate that the extent of depletion of structural nucleoporins can lead to distinct graded phenotypes in zebrafish, and that the kidney appears to be the organ with the highest sensitivity to moderate *Nup133* or *Nup107* deficiencies.

As we submitted our manuscript, the only Y-complex nucleoporin gene found to be mutated in SRNS was *Nup107*^15–18^. In addition, a study had revealed that normal expression level of another Y-complex component, Nup160, is critical for the proliferation and viability of podocytes *in vitro*^43^. Because our data indicated that Nup133, the direct partner of Nup107 within the Y-complex, is also required for proper glomerular function in zebrafish, we proposed that in addition to *Nup107* at least these two other Y-Nups, or possibly all the nine Y-complex subunits (*Nup107, Nup133, Nup160, Nup96, Nup85/75, Nup43, Nup37, Seh1*, and *Sec13*) were candidate genes that would be worth testing in SRNS patients. While our manuscript was under revision, two publications came out that confirmed our hypothesis: specific hypomorphic mutations in 4 Y-complex components, namely Nup107, Nup133, Nup160 and Nup85 were reported to cause SRNS^42^ whereas a homozygous splicing mutation in *NUP133* was identified in a GAMOS family with SRNS combined to brain atrophy^40^. Noteworthy, the mutation found in this GAMOS family caused reduced Nup133 protein level^40^, as also previously described for the consanguineous families with homozygous mutation in *NUP107* and a GAMOS-like presentation^18^. Although Nup133 protein levels were not investigated in patients solely featuring SRNS, the data obtained in zebrafish (our study and^40^) suggest that more severe alteration leading to decreased expression or stability of Nup133 would cause neuronal in addition to renal defects.

Several mechanisms could explain the implication of Nup133, and more generally, of Y-complex Nups, in glomerular function and thus NS. While it was recently reported that alterations of several Y-Nups including *NUP133* causes dysregulation of Cdc42 activity in human podocytes, the authors suggested that this was likely an indirect effect, possibly linked to altered nucleocytoplasmic transport^42^. Our immunofluorescence data using mAb414 did not reveal any major alteration of overall NPC assembly in *nup133* morphants. However, Nup133 deficiency may alter the nuclear transport of specific molecules, as for instance reported for Nup93 that belongs to another structural domain of the NPC^14^. Indeed, human podocytes and HEK293 cells expressing Nup93 mutations identified in SRNS patients exhibited impaired nuclear import of SMAD4 upon stimulation with bone morphogenetic protein 7 (BMP7), a secreted molecule involved in kidney development and response to renal injury. This result was consistent with a previous study in which depletion of Drosophila Nup93 led to a defective import of the SMAD4 orthologue^44^. Noteworthy, the latter study also revealed the implication of two Drosophila Y-Nups, Nup85/75 and Sec13, but not of Nup133, in this specific import pathway^44^. While we cannot formally exclude Nup133 contribution to the nuclear import of SMAD4 in vertebrates, Nup133 depletion may also affect other signaling cascades required for the maintenance of an intact GFB. Indeed, two Y-complex subunits, Nup107 and Nup37, were reported to be regulators of the ERK and YAP pathways, respectively^45,46^.

Nup133 knockdown may also alter nuclear mechanotransduction, for instance by interfering with the ‘linker of nucleoskeleton and cytoskeleton’ (LINC) complex that establish a stable and cross-linked network between the cytoskeleton and the lamina underneath the NE (reviewed in^47^). Indeed, while Nup133 is required for the proper assembly of the NPC basket^48^, intimate links between the NPC basket, the LINC subunit Sun1 and Lamin-C have been reported^49,50^. As previously discussed^16^, deregulation of mechanotransduction signaling pathways may in turn affect podocytes that are subjected to mechanical stretching caused by capillary pressure. As reported for a subset of nucleoporins (reviewed in^51^), Nup133 may also be involved in gene regulation and thus modulate the expression of specific genes required for proper function of the GFB, notably in podocytes.

Finally, one may also keep in mind that several nucleoporins localize at the base of the cilia^52,53^. In particular, another member of the Y-complex, Nup85, was recently reported to be required for proper cilia localization of Nup98, a nucleoporin that regulates diffusion of soluble molecules through the ciliary base^54^. Moreover, mutations affecting the kinetochore protein Cenp-F, an established partner of Nup133^55^, were identified in patients affected by severe ciliopathy and microcephaly^56^. Because the appearance of kidney cysts in zebrafish can also be caused by inactivation of several ciliopathy genes (reviewed in^57,58^), the observation of a mild Left-Right patterning defect in *nup133* morphants was noteworthy. However, our data revealed that unlike the glomerular cysts phenotype, the Left-Right patterning defect is not rescued by the *3xHA-mCherry-Dr nup133* transgene, and is therefore not correlated with the glomerular defect observed in these larvae. As previously proposed for other mutants^37,59,60^), the pronephric cyst phenotype observed in the *nup133* morphants might thus be primarily caused by alterations of the glomerular filtration barrier and the subsequent inability to osmoregulate, rather than the indirect consequence of cilia defect. While this non-rescued *situs inversus* phenotype may reflect an off-target effect of the morpholinos (as previously reported in another study)^61^, we do not formally rule out a possible impact of Nup133 depletion on ciliary function. The lack of rescue of the *situs inversus* by our *nup133* construct would then reflect an improper expression of the transgene in the Kupffer’s vesicle (the ciliated organ that initiates left-right development in the zebrafish embryo)^62^. Analysis of a potential cell-type-dependent ciliary function of Nup133 will deserve a full and independent study.

In conclusion, our study on zebrafish *Nup133*, together with an increasing number of recent studies correlating single-gene mutations in *Nups* with cell type-specific defects, strengthens the emerging concept of specific “nucleoporopathies”^1^, an exciting development in the nuclear pore complex field.

## Materials and Methods

### Fish maintenance and breeding

Zebrafish (*Danio rerio*) were kept at 26 °C under a 14-h/10-h light/dark cycle and bred as previously described^63^. Larval stages were raised at 28 °C in E3 embryo medium (5 mM NaCl, 0.17 mM KCl, 0.33 mM CaCl_2_, 0.33 mM MgSO_4_) containing 0.01% methylene blue. 0.003% PTU (1-phenyl-2-thiourea; Sigma Aldrich) was added in embryo medium to inhibit melanin synthesis during larval development and facilitate fluorescent microscopy. *Tg(wt1b:EGFP)* line was a kind gift from Dr. Christoph Englert (Leibniz Age Research, Jena, Germany). All experiments were performed in accordance with internationally recognized and with Swiss legal ethical guidelines for the use of fish in biomedical research and experiments were approved by the local authorities (Veterinäramt Zürich Tierhaltungsnummer 150 and TV4206).

### Whole-mount *in situ* hybridization

Sequences were identified and annotated using combined information from expressed sequence tags and genome databases (GeneBank, http://www.ncbi.nlm.nih.gov; Ensembl, http://www.ensembl.org/index.html). The primers used for probe preparation are listed in supplementary Table S1. *nup133, pax2a, wt1a* and *cdh17* cDNAs were all isolated by RT-PCR from total RNA from 24–48 hpf embryos and cloned into TOPO pCRII vector (TA Cloning Kit Dual Promoter, Invitrogen) as previously described^64^. The resulting plasmids were linearized for SP6 and T7 *in vitro* transcription and purified with phenol-chloroform. Digoxigenin (DIG)-labeled antisense (or sense, as negative control for *nup133* expression) RNA probes were generated using DIG-RNA-labeling kit (Roche Diagnostic). Zebrafish embryos were fixed in 4% paraformaldehyde in phosphate-buffered saline (PBS) at 4 °C overnight and whole mount *in situ* hybridization was performed as previously described^65^. Briefly, on day 1 the larvae were treated with proteinase K and then fixed with 4% paraformaldehyde (PFA) before prehybridization at 64 °C. Hybridization of RNA probes was done at 64 °C overnight. On day 2, after several stringency washes at 64 °C, probes were blocked in 1 × Roche blocking solution in Tris/NaCl/Tween. Anti-DIG AP antibody was applied overnight at 4 °C. On day 3, after several washing steps, signal was detected by incubation in staining buffer. Stained embryos were postfixed with PFA overnight and imaged in glycerol with an Olympus BX61 light microscope or with a stereomicroscope (Olympus MVX10). Following PFA postfixation, 4 and 5 dpf embryos were cryoprotected in 30% sucrose overnight and embedded in tissue freezing medium (Richard-Allan Scientific Neg-50 Frozen Section Medium Thermo Fisher Scientific). 14–16 μm transverse sections were cut on a Microm HM 550 cryostat and imaged with a Olympus BX61 wide-field microscope (Volketswil, Switzerland). Images were processed and assembled using Adobe Photoshop and Adobe Illustrator CS6.

### Morpholino and mRNA injections

The *nup133* gene was targeted with specific antisense splice-blocking Morpholinos (MOs) (GeneTools, Philamath, OR) designed to target the exon-intron boundary of exon 3 (splice donor, *nup133*_E3I3) and the intron-exon boundary of exon 4, respectively (splice acceptor, *nup133*_I3E4) (MO sequences are provided in Supplemental Table S1). The morpholinos were diluted in RNase-free water with 0.1% phenol red as an injection tracer and ~1 nl was injected into fertilized embryos at 1–2 cell stage. Uninjected sibling embryos and embryos injected with standard control morpholinos were used as controls. Evaluation of morphological changes was performed at 3 dpf. A range of concentration was first used to determine the optimal MO amount required to induce a specific phenotype without inducing toxicity (Supplemental Fig. S3). The two MOs were then always injected at 1.75 ng each.

For the rescue experiments, a pBluescript KSM vector containing *3xHA-mCherry-Dr nup133* was used. The plasmid was generated using PCR amplification using proofreading DNA polymerases (Phusion HF, NEB) and NEBuilder HiFi DNA Assembly Cloning Kits. Briefly, the sequence encoding three copies of the influenza virus *hemagglutinin* (*HA*) epitope was amplified from pFA6a-3HA-kanMX6^66^, the *mCherry* was amplified from plasmid #1937^48^, full-length zebrafish *nup133* was amplified from a Dharmacon cDNA vector (Clone ID: 2600558) and the three PCR products were inserted by recombination in a pBluescript KSM vector (Stratagene). PCR-amplified fragments and junctions were checked by sequencing. Plasmid map is available upon request. For *in vitro* mRNA production the *3xHA-mCherry-Dr nup133* coding fragment was linearized by digestion with the restriction enzyme NotI and purified with phenol-chloroform extraction. Capped and tailed RNA was *in vitro* transcribed using the mMessage mMachine T3 kit (Life Technologies, Zug, Switzerland) according to the manufacturer’s instruction. A polyA tail was added with polyA tailing kit (Invitrogen by Thermo Fischer Scientific) followed by purification with MEGAclear Transcription Clean-Up Kit (Ambion). The mRNA (300 nM) was injected into the embryos at one cell stage before MO injection.

### Real-time PCR and real time quantitative PCR

To examine splicing defects caused by *nup133*_E3I3 and *nup133*_I3E4 injection, total RNAs were extracted from 10–40 control or MO-injected embryos at 1 dpf by using the ReliaPrep RNA Tissue Miniprep System (Promega), and RNAs were reverse transcribed and amplified using SuperScript III First-Strand Synthesis SuperMix with random hexamers (Invitrogen). The resulting cDNAs were then characterized by RT-PCR using primers designed from flanking exon-coding sequence (Fig. 2a,b and Supplementary Table S1). The RT-PCR products were purified from gel, re-amplified with nested primers and altered splicing was confirmed by sequencing.

Real-time quantitative PCR was performed on independent batches of cDNAs from 1, 2 or 5 dpf embryos with a LightCycler480 instrument (Roche Life Sciences) by using SYBR Green incorporation (SYBR Green PCR-Master Mix; Applied Biosystems) and specific primer pairs (listed in Supplementary Table S1). The relative amounts of cDNAs in the samples were quantified according to the manufacturer’s instructions and normalized by reference to the actin beta 2 (*actb2*) cDNAs.

### Whole mount live-animal imaging

To evaluate morphological changes, larvae were anesthetized with 200 mg/ml 3-aminobenzoic acid methyl ester (MESAB, Sigma-Aldrich), mounted in 1.5% low melting temperature agarose in E3 medium and imaged using a stereomicroscope (Olympus MVX10) equipped with a color camera (ColorViewIII, Soft imaging System, Olympus).

### Western blotting

For western blot analyses, groups of 20–40 embryos at 24 hpf were homogenized by sonication in 50–100 µl of Laemmli buffer. The lysates were separated either on 4–20% Mini-Protean® TGX Stain-Free™ gel or on NuPAGE^TM^ 4–12% Bis-Tris gels, using Tris-Glycine and MOPS as running buffer, respectively, and transferred to nitrocellulose membranes. The resulting blots were stained using Ponceau, saturated with TBS, 0.1% Tween, and 5% dried milk and probed overnight at 4 °C with Nup133 antibody (Rabbit monoclonal [EPR10809] to NUP133, ab181355; Abcam, 1:500), rabbit polyclonal anti-ß-Actin (Cell Signaling Technology, #4967; 1:1000), or mouse monoclonal antibody HA.11 (clone 16B12; Eurogentec #MMS-101R; 1:2,000). Incubations of the membrane with primary and HRP-conjugated secondary antibodies (Jackson ImmunoResearch Laboratories) were done in TBS buffer (0.1% Tween, 5% dried milk), and signals were detected by enhanced chemiluminescence (SuperSignal® West Femto or Pico PLUS; Thermo Scientific).

### NPC labeling on cryosections

Because the glomerular phenotype is not fully penetrant (observed, depending on the experiment, in 40–90% of the injected embryos, Supplemental Fig. S4), only *nup133* MO embryos displaying kidney cysts (visualized using the *wt1b:GFP* transgene) were analyzed. For mAb414 staining, 3dpf zebrafish larvae were fixed for 30 min in 3% PFA, cryoprotected in 30% sucrose and embedded in tissue freezing medium (OCT - Leica 14020108926). Following freezing, 14 μm transverse sections were cut on a Leica CM3050S cryostat and placed on SuperFrost Ultra Plus™ adhesion slides (Thermo Scientific). The slides were dried for 30 min at 37 °C, washed for 5 min in PBS, saturated for 30 min with PBS containing 0.1% Triton X-100 and 1% BSA (PBS-T-BSA) and incubated overnight at 4 °C with mAb414 mouse monoclonal antibody (Covance, MMS-120P) diluted at 1/5000 in the same buffer. Following rapid washes in PBS-T, slides were then incubated for 2 hours at room temperature with CY3-conjugated anti-mouse antibody (Jackson ImmuoResearch, # 715-165-151) diluted 1/500 in PBS-T-BSA containing 0.1 µg/ml DAPI. The slides were then washed in PBS-T and mounted in Moviol medium.

Spinning disk images were acquired on an inverted microscope (DMI8; Leica) with a CSU-W1 spinning disk head (Yokogawa) and a sCMOS ORCA-Flash 4 V2+ camera (Hamamatsu) using 100×/1.4 oil objective or a 20×/0.75 oil objective. Images were acquired without camera binning using MetaMorph (Universal Imaging Corp.), processed using ImageJ/Fiji software and assembled using Adobe Photoshop CS. A unique plane is presented.

### Glomerular filtration assay

Zebrafish larvae at 4 dpf were anesthetized with 200 mg/ml 3-aminobenzoic acid methyl ester (MESAB, Sigma-Aldrich). 1 nl of Albumin from bovine serum (BSA), Alexa Fluor 647 conjugate (Thermo Fischer scientific) was injected into the common cardinal vein according to^38^. The larvae were transferred to E3 medium for recovery. 20 mins after injections, the larvae were fixed in 4% PFA overnight and processed for cryosections and imaging.

### Transmission electron microscopy and histology

Zebrafish larvae at 5 dpf were fixed overnight in 2.5% glutaraldehyde (along with 3% EM grade PFA for the larvae shown in Fig. 7h and in Supplementary Figs. S6, panels b, d and e, and S7, panel f) in 0.1 M cacodylate buffer, pH 7.2. As indicated above for the IF studies, *nup133* MO embryos displaying kidney cysts were selected prior to EM studies. To achieve a better penetration of the fixative, the tail of each larva was cut off with a scalpel prior to fixation. The larvae were rinsed in 0.1 M cacodylate buffer before postfixation in 1% osmium tetroxide in cacodylate buffer for 1 hour at room temperature. The samples were rinsed in distilled water before en block staining in 1% aqueous uranyl acetate for 1 hour at room temperature. Following dehydration through a graded series of ethanol ranging from 50% to 100%, the larvae were infiltrated overnight in 66% Epon/Araldite in propylene oxide. Finally, the specimens were embedded in 100% Epon/Araldite and placed in a polymerizing oven at 60 °C for 26 h. Semi thin section (2 µm) were stained with toluidine blue and used for histological studies. Ultrathin sections (65 nm) of the glomerulus and the proximal tubules, obtained using a Leica EM FCS ultramicrotome were collected on formvar coated copper grids, stained in lead for 5 minutes to increase the contrast, and examined with a Philips CM-100 scope at 80 kV. Images were acquired using the Gatan Microscopy Software.

For quantification of FP density, images acquired from 3 control and 3 *nup133*-MO treated embryos were analyzed. For each embryo, the number of foot processes and the length of the basement membrane were measured from 3–5 distinct fields each. Images used for quantification, the selected ROI, and original Excel file used to generate the graph are available at Mendeley under 10.17632/j78ddshctz.1.

Statistical analyses were performed using Wilcoxon-Mann-Whitney Rank Sum Test provided by the KaleidaGraph software.

## Supplementary information

Supplementary information

## Data Availability

pBluescript KSM vector containing *3xHA-mCherry-Dr nup133* and its sequences are available upon request. Images used for EM quantification (Fig. 7g) and for the quantifications of kidney cysts appearance, LR asymmetry (based on pancreas positioning) and head sizes (Supplemental Fig. S5) are available at Mendeley under 10.17632/j78ddshctz.1.
