# Author Correction: Moderate Nucleoporin 133 deficiency leads to glomerular damage in zebrafish

**DOI:** 10.1038/s41598-020-57829-7

**Published:** 2020-01-15

**Authors:** Chiara Cianciolo Cosentino, Alessandro Berto, Stéphane Pelletier, Michelle Hari, Johannes Loffing, Stephan C. F. Neuhauss, Valérie Doye

**Affiliations:** 1 Institute of Molecular Life Sciences, University of Zurich, Zurich, Switzerland; 2 Institut Jacques Monod, UMR7592 CNRS-Université Paris Diderot, Sorbonne Paris Cité, F-75205 Paris, France; 3 Ecole Doctorale SDSV, Université Paris Sud, F-91405 Orsay, France; 4 Institute of Anatomy, University of Zurich, Zurich, Switzerland; 5 Fondazione RiMED, Palermo, Italy

Correction to: *Scientific Reports* 10.1038/s41598-019-41202-4, published online 20 March 2019

This article contains an error in the Supplementary Information file in Supplementary Table S1, where the sequence of the I3E4 morpholino contains a duplicated base:

“CGAACAGCTGAACGGCAAAATAAAAC”

should read:

“CGACAGCTGAACGGCAAAATAAAAC”

