# Publisher Correction: Moderate Nucleoporin 133 deficiency leads to glomerular damage in zebrafish

**DOI:** 10.1038/s41598-020-58959-8

**Published:** 2020-02-19

**Authors:** Chiara Cianciolo Cosentino, Alessandro Berto, Stéphane Pelletier, Michelle Hari, Johannes Loffing, Stephan C. F. Neuhauss, Valérie Doye

**Affiliations:** 1 Institute of Molecular Life Sciences, University of Zurich, Zurich, Switzerland; 2 Institut Jacques Monod, UMR7592 CNRS-Université Paris Diderot, Sorbonne Paris Cité, F-75205 Paris, France; 3 Ecole Doctorale SDSV, Université Paris Sud, F-91405 Orsay, France; 4 Institute of Anatomy, University of Zurich, Zurich, Switzerland; 5 Fondazione RiMED, Palermo, Italy

Correction to: *Scientific Reports* 10.1038/s41598-019-41202-4, published online 20 March 2019

The original PDF version of this Article contained a typographical error in the publication date ‘20th March 2019’ which was incorrectly given as ‘18th March 2019’. This has now been corrected in the PDF version of the Article. The HTML version was correct at the time of publication.

