# Supplementary material for: Moderate Nucleoporin 133 deficiency leads to glomerular damage in zebrafish
Source: Sci Rep. 2019 Mar 20;9:4750. doi: 10.1038/s41598-019-41202-4 (PMC6426968; doi:10.1038/s41598-019-41202-4)
Supplement: Supplementary file 1 — Supplementary information [file 41598_2019_41202_MOESM1_ESM.pdf]

## SUPPLEMENTARY INFORMATION

# Moderate Nucleoporin 133 deficiency leads to glomerular damage in zebrafish

Chiara Cianciolo Cosentino[1,4,5]*, Alessandro Berto[2,3]*, Stéphane Pelletier[2], Michelle Hari[1], Johannes Loffing[4], Stephan C. F. Neuhauss[1], and Valérie Doye[2@]

[1] Institute of Molecular Life Sciences, University of Zurich, Zurich, Switzerland

[2] Institut Jacques Monod, UMR7592 CNRS-Université Paris Diderot, Sorbonne Paris Cité, F-75205 Paris, France

[3] Ecole Doctorale SDSV, Université Paris Sud, F-91405 Orsay, France.

[4] Institute of Anatomy, University of Zurich, Zurich, Switzerland

[5] Fondazione RiMED, Palermo

*equivalent contribution

[@] Corresponding author:
Tel: + 33 1 57 27 80 60
Fax: +33 1 57 27 80 63
Email: valerie.doye@ijm.fr

```
human          22 GLGPGSTPRTASRKGLPLGSAVSSPVLFSPVGRRSSLSSRGTPTRMFPHHSITESVNYDVKTFGSSLPVKVMEALTLAEVDDQLTINIDEGGWACLVCKE    121
                  |.|....|||..|:.:   |||...:||||  |||::::|.||||: ..|::.||.|:||:|||||||||||||||:|:||||:::.::..|||.:||.|
zebrafish       9 GSGRRQAPRTGGRRSV---SAVQPGLLFSP--RRSAVTARSTPTRV-QSHAVVESYNFDVQTFGSSLPVKVMEALTMADVDDQISVKVEASGWAWMVCGE    102

human         122 KLIIWKIALSPITKLSVCKELQLPPSDFHWSADLVALSYSSPSGEAHSTQAVAVMVATREGSIRYWPSLAGEDTYTEAFVDSGGDKTYSFLTAVQGGSFI    221
                  :||:||::.:.:.||||||:||||.|:|.:|||||::|.|.|...| ..|:::|:..:.:|.:|:|||||.|.:|||..:|..|..: :::.||:|||||
zebrafish     103 RLIVWKVSQTSVAKLSVCKDLQLPSSEFAYSADLVSISSSGPLDLA-PIQSISVLAVSPDGLVRFWPSLAHEGSYTEISLDLSGHLS-NYVAAVKGGSFI    200

human         222 LSSSGSQLIRLIPESSGKIHQHILPQGQGMLSGIGRKVSSLFGILSPSSDLTLSSVLWDRERSSFYSLTSSNISKWELDDSSEKHAYSWDINRALKENIT    321
                  :||....|:||..:||||:|...:.|||||||||||:|||||||....:||::.||||.:..|..|||:|..:||||:|::||....||..|:.:.::||
zebrafish     201 VSSYRGHLLRLSADSSGKLHHRPVQQGQGMLSGIGRRVSSLFGIRGQPADLSVFSVLWVKASSCLYSLSSCGLSKWEVDENSETQVLSWSTNQIITDSIT    300

human         322 DAIWGSESNYEAIKEGVNIRYLDLKQNCDGLVILAAAWHSADNPCLIYYSLITIEDNGCQMSDAVTVEVTQYNPPFQSED-LILCQLTVPNFSNQTAYLY    420
                  ||||.|||||..||:|||:.|||::.:..|||:|||||:..|.||:.|:.|:|:.::.....|.:|||||:||||||||: |:..:|.:|:.|:..||||
zebrafish     301 DAIWDSESNYSEIKKGVNVLYLDMQPSNAGLVVLAAAWYPGDTPCVAYFCLVTLAESIVPSPDLLTVEVTKYNPPFQSEEELLKTRLVLPDPSSPAAYLY    400

human         421 NESAVYVCSTGTGKFSLPQEKIVFNAQGDSVLGAGACGGVPIIFSRNSGLVSITSRENVSILAEDLEGSLASSVAGPNSESMIFETTTKNETIAQEDKIK    520
                  ||..|:.||||.|:..|.:|||:|::.|||.|.|.|.|..:|:.||:|||||::.:||..|:|.|.:|.||.:|||||..|....||..|.:.:|||||.|
zebrafish     401 NEELVFACSTGAGRGGLAEEKILFSSPGDRVRGGGVCADLPVFFSQNSGLVAVLARETASLLPETMEDSLCTSVAGPGPEGTPLETPPKIDMVAQEDKTK    500

human         521 LLKAAFLQYCRKDLGHAQMVVDELFSSHSDLDSDSELDRAVTQISVDLMDDYPASDPRWAESVPEEAPGFSNTSLIILHQLEDKMKAHSFLMDFIHQVGL    620
                  |||.||||:||.||..||.:|||||.|  |.:..::||..||||.:||:|||||.|||||||||:|..||:.||||:|||||||||||..||||:.|.||
zebrafish     501 LLKQAFLQFCRHDLVGAQSMVDELFPS--DGEGSADLDTVVTQIDLDLVDDYPACDPRWAESVPDEGAGFTLTSLILLHQLEDKMKAHRCLMDFLLQTGL    598

human         621 FGRLGSFPVRGTPMATRLLLCEHAEKLSAAIVLKNHHSRLSDLVNTAILIALNKREYEIPSNLTPADVFFREVSQVDTICECLLEHEEQVLRDAPMDSIE    720
                  ..||.|..||..|||||||||||||||||||||||||::..:||||||..||.|...:.|:|||||||||||||:.:|.||||:.||:.|::.| |:..
zebrafish     599 LDRLTSTKVRSCPMATRLLLCEHAEKLSAAIVLKNHHAKHPELVNTAIQTALKKNSTDTPTNLTPADVFFREVSQISSIFECLLDEEEKALKEHP-DAAR    697

human         721 WAEVVINVNNILKDMLQAASHYRQNRNSLYRREESLEKEPEYVPWTATSGPGGIRTVIIRQHEIVLKVAYPQADSNLRNIVTEQLVALIDCFLDGYVSQL    820
                  |.|||::||:|:|||||||:.||:.:.||||..|:...||||:||||:.|.||:|:||.||||::|:.|||.||:.||:::.||||||:|..|.|||:||
zebrafish     698 WGEVVLSVNDIIKDMLQAAAQYRETKASLYRAPENCSPEPEYIPWTASGGVGGVRSVISRQHELILRAAYPHADAELRSVLCEQLVALLDSLLSGYVAQL    797

human         821 KSVDKSSNRERYDNLEMEYLQKRSDLLSPLLSLGQYLWAASLAEKYCDFDILVQMCEQTDNQSRLQRYMTQFADQNFSDFLFRWYLEKGKRGKLLSQPIS    920
                  .|:.:...:||||.||.||.||||:||.|||.|||:.|.|:|||||||||||||:||:||||||||:||.:||||||:|||||:||||||||||||||::
zebrafish     798 TSLRRGGQQERYDTLENEYTQKRSELLKPLLELGQHQWVAALAEKYCDFDILVQLCERTDNQSRLQQYMVKFADQNFADFLFRWYMEKGKRGKLLSQPMA    897

human         921 QHGQLANFLQAHEHLSWLHEINSQELEKAHATLLGLANMETRYFAKKKTLLGLSKLAALASDFSEDMLQEKIEEMAEQERFLLHQETLPEQLLAEKQLNL   1020
                  .|.|||:|||||:||||||:|:.|:.::||.||...||||||||:||||||.||||.|||||..|.:.:.::.::.|||||||||||||:|||.|||||.
zebrafish     898 THQQLASFLQAHDHLSWLHDIHVQDYQRAHRTLYNQANMETRYFSKKKTLLALSKLTALASDMPEPVHRRQLNDIVEQERFLLHQETLPKQLLEEKQLNP    997

human        1021 SAMPVLTAPQLIGLYICEENRRANEYDFKKALDLLEYIDEEEDININDLKLEILCKALQRD---NWSSSDGKDDPIEVSKDSIFVKILQKLLKDGIQLSE   1117
                  .:||:|:...||.||||:|||.|||||||||||||||.:||..|:::.||.||..|||::|   :|||||..|||:|.::||.||||||||:::.:.|..
zebrafish     998 DSMPLLSPQNLISLYICDENRGANEYDFKKALDLLEYFEEENGIDVDALKREIFSKALKKDWKESWSSSDDNDDPLEAARDSTFVKILQKLIQERVSLQT   1097

human        1118 YLPEVKDLLQADQLGSLKSNPYFEFVLKANYEYYVQGQI   1156
                  |||::|||||.|:|.||||.|||||:|:||||:|::.||
zebrafish    1098 YLPDIKDLLQEDELESLKSKPYFEFLLRANYEHYLKVQI   1136
```

**Supplementary Figure S1. Alignment of human and zebrafish Nup133 proteins.** Alignments between human and zebrafish Nup133 (UniProtKB Reference Sequence: Q8WUM0 (NU133_HUMAN) and F1QNV4 (F1QNV4_DANRE), respectively) were performed based on EMBOSS Matcher. The human amino acid sequence highlighted in grey indicates the region that folds in a β-propeller [1] while the yellow sequence folds in a α-solenoid [2]. In bold are indicated from N- to -C the binding site of mouse Nup133 for Cenp-F [3], the ALPS motif [4] and the binding site for Nup107 [2].

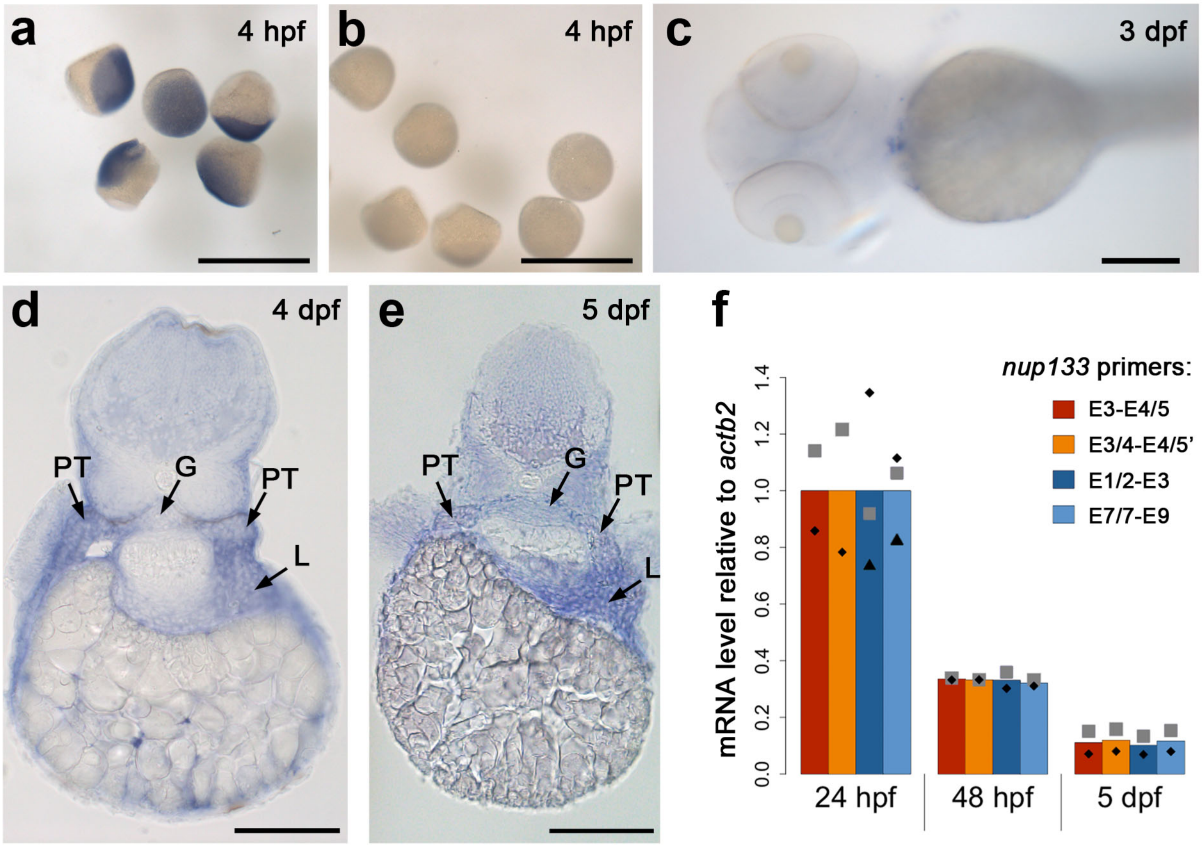


**Supplementary Figure S2. Analysis of *nup133* expression in zebrafish embryos by in situ mRNA hybridization (ISH) and quantitative RT-PCR (RT-qPCR).**

Zebrafish embryos were hybridized with antisense (**a, d, e**) or sense (**b, c**) *nup133* mRNA probes. Whole mount ISH at sphere stage (**a**, **b**, scale bars 1 mm) and 3 dpf (**c**, scale bar 200 µm), and transverse sections of 4 and 5 dpf embryo at the level of the pectoral fins (**d, e**, scale bars, 100 µm) are presented. (**f**) *nup133* mRNA levels relative to *actb2* expression was determined by RT-qPCR on 24 hpf, 48 hpf and 5 dpf embryos using 4 distinct primer pairs (listed in Supplementary Table S1 and positioned above the scheme of nup133 exon structure in Figure 2a). For each pair of primers, the mean of 2-3 distinct experiments (each represented by a distinct symbol) was set to 1 at 24 hpf.

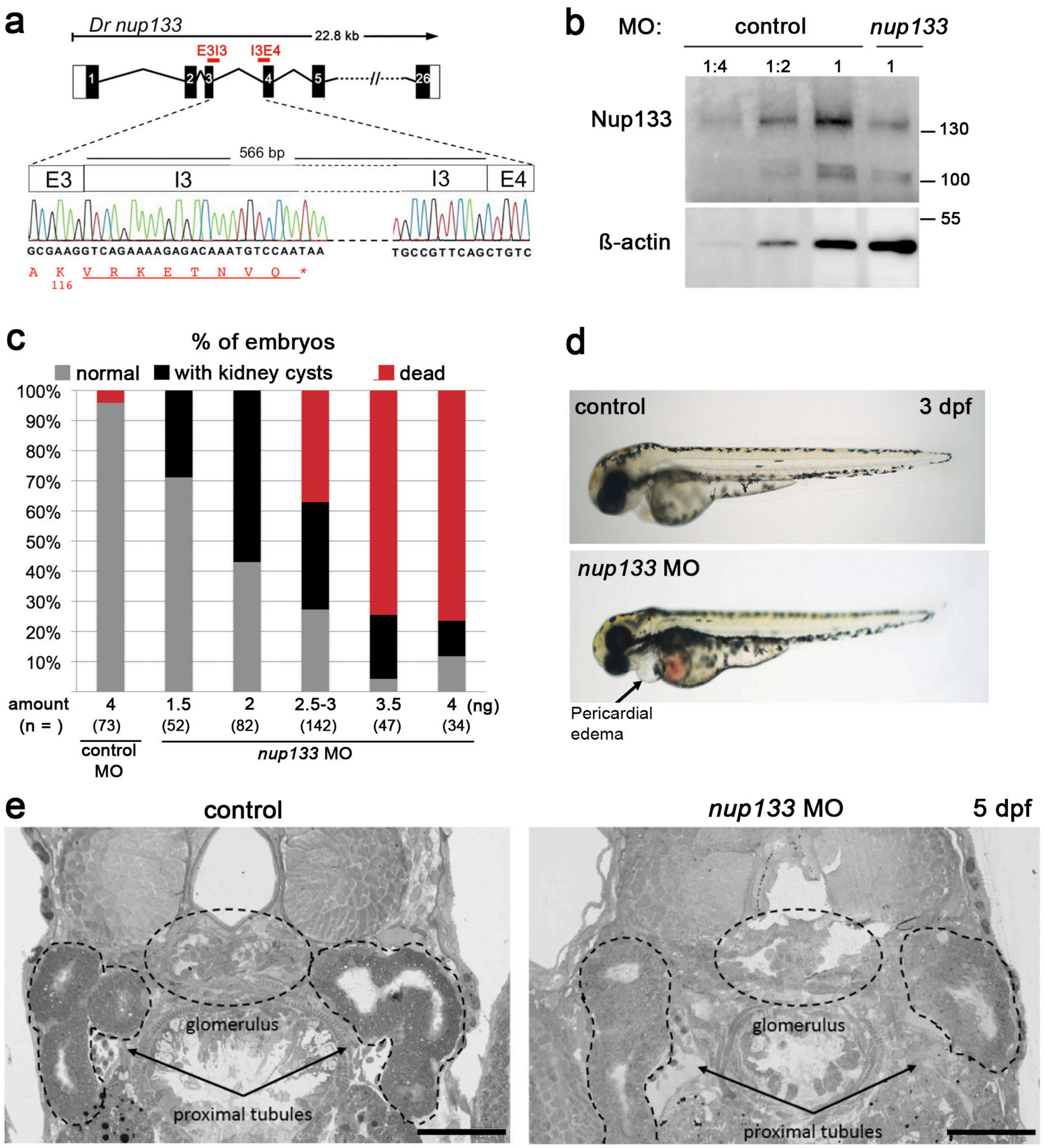


**Supplementary Figure S3. Retention of intron 3 in *nup133* MO injected embryos and morphology of 3 and 5 dpf control and *nup133* MO larvae.** (**a**) Exon structure of *Danio rerio* (*Dr*) *nup133* around the binding sites of the E3I3 and I3E4 splice morpholinos. The size of intron 3 is indicated. Sequencing of the additional RT-PCR product in the *nup133*MO embryos shows retention of intron 3. The end of the predicted aa sequence of the corresponding truncated protein is indicated in red, under the DNA sequence. Residues encoded by the intron are underlined. (**b**) Extracts from 24 hpf control and *nup133* MO embryos were analyzed by western blot using anti-Nup133 and anti ß-actin antibodies (used as loading control). Dilutions of the control samples (1:2 and 1:4) were also loaded to better appreciate the decrease of Nup133 protein level. (**c**) Relative proportions of 3dpf embryos with kidney cysts or dead upon injection of the indicated amounts of control or of each *nup133* MO. For each condition, the total number of embryos analyzed is indicated (n=). (**d**) Gross morphology of 3 dpf control and *nup133* MO larvae. Note the pericardial edema in *nup133* MO larvae (arrow). (**e**) Toluidine blue stained sections of 5 dpf uninjected control (left panel) and *nup133* MO injected larvae (right panel) showing the glomerulus and the proximal tubules (indicated with dotted lines). Note the cystic dilation of the glomerulus in the *nup133* MO larvae that is not associated with a major dilatation of the proximal tubules. Scale bars 50 µm.

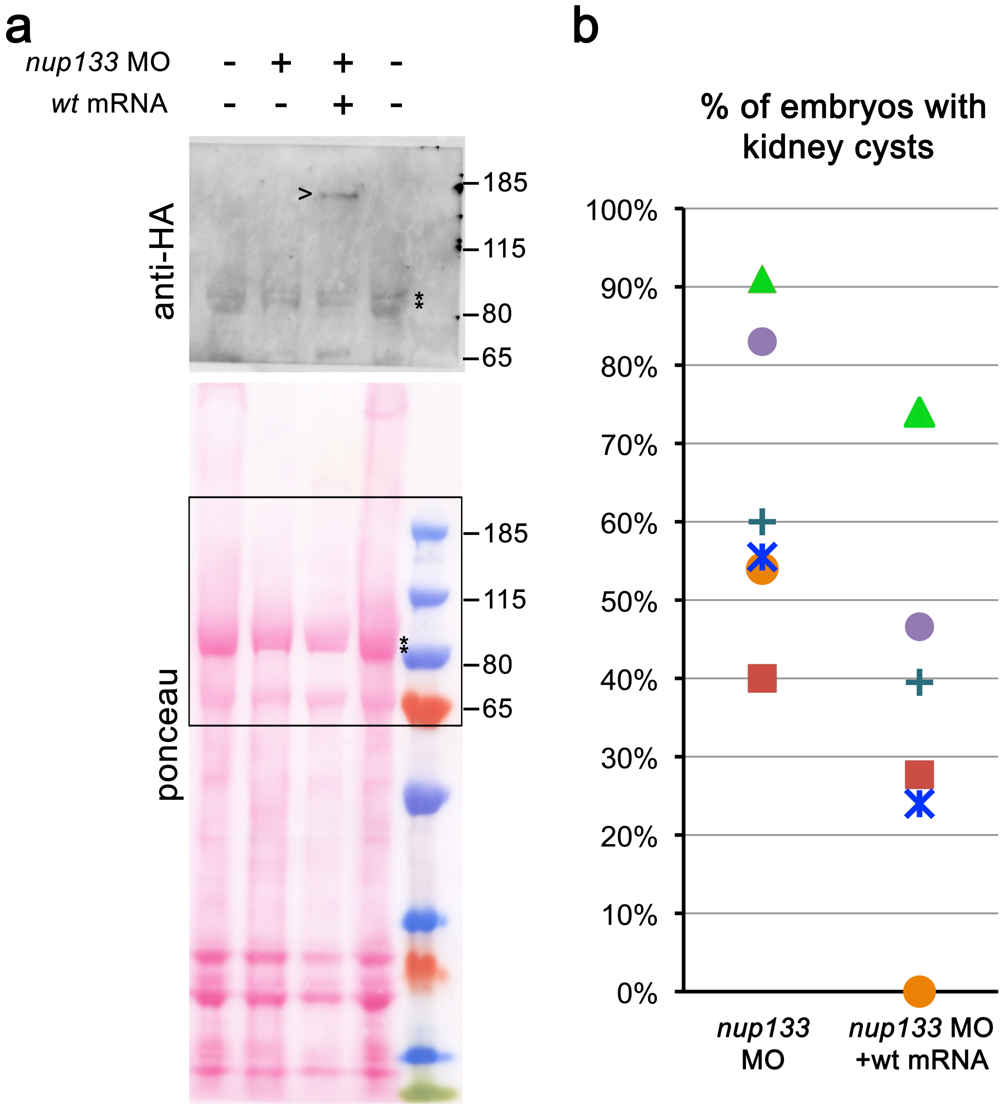


**Supplementary Figure S4. Expression of 3xHA-mCherry-Dr Nup133 rescues the *nup133* MO phenotype**

(**a**) Extracts from 24 hpf embryos were analyzed by western blot using anti-HA antibodies. The ponceau staining of the entire nitrocellulose membrane is also shown, and the square indicates the area that was hybridized with the anti-HA antibody. The arrowhead points to the position of the 3xHA-mCherry-Dr Nup133. Stars indicate abundant proteins in the extract that are non-specifically recognized by the anti-HA antibody. **(b)** Percentage of embryos showing kidney cysts at 3 dpf. For each of the 5 independent experiments (combined in Fig. 2e), the same symbols are used for embryos that were or not injected with *3xHA-mCherry-Dr nup133* mRNA (+ wt mRNA) prior to injection with *nup133* MO.

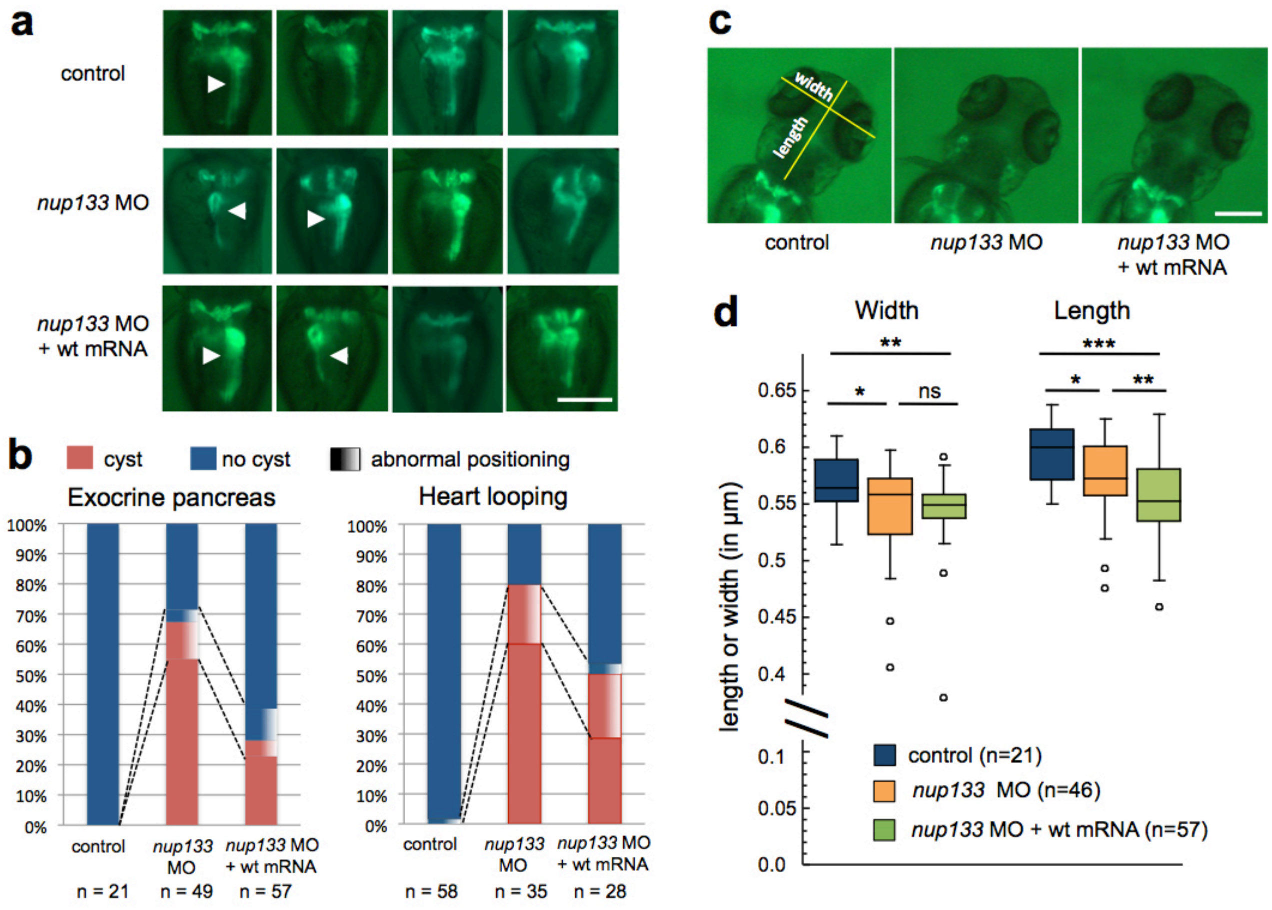


**Supplemental Figure S5. Unlike the appearance of kidney cysts, the Left-Right patterning defects and the mild alteration of head size observed in *nup133* morphants are not rescued by wt *nup133* mRNA.** (**a, c**) Dorsal view of (**a**) the bodies and (**c**) the heads of 3dpf *Tg(wt1b:EGFP)* embryos injected with control or *nup133* MO, or sequentially injected with *3xHA-mCherry-Dr nup133* mRNA and *nup133* MO (*nup133* MO + wt mRNA). Overlays of transmission and GFP-signal images are presented. In (**a**), arrowheads point to the exocrine pancreas that is located on the right side of control larvae, but is sometimes misplaced towards the left side in *nup133* MO injected embryos, injected or not with *3xHA-mCherry-Dr nup133* mRNA. (**b**) The presence of glomerular cysts and either the abnormal positioning of the exocrine pancreas (left panel) or heart looping (right panel) were recorded on the indicated number of 3 dpf and 2 dpf larvae, respectively. The exocrine pancreas was scored as normal, when positioned to the right and as "abnormal positioning" when positioned to the left or in the midline. Heart looping was scored as normal if the ventricle was on the right of the atrium (D-looping), and as "abnormal positioning" if the ventricle was either on the left of the atrium (L loop) or if it failed to bend (no-loop). In (**c**), the yellow lines on the control larva corresponds to the lines drown in Image J to measure the width and length of embryos heads. (**d**) Length and width of the heads were measured from the indicated number of control, *nup133* MO and *nup133* MO+wt mRNA larvae. Box plots were generated using KaleidaGraph (Synergy Software): each box encloses 50% of the normalized values obtained, centered on the median value. The bars extending from the top and bottom of each box mark the minimum and maximum values within the dataset falling within an acceptable range. Values falling outside of this range are displayed as an individual point. Statistical analyses were performed using Wilcoxon-Mann-Whitney Rank Sum Test provided by the KaleidaGraph software. Standard conventions for symbols indicating statistical significance are used: ns, not significant: $P > 0.05$; *:$P \leq 0.05$, ** :$P \leq 0.01$, *** :$P \leq 0.001$. Images and regions used for quantification of exocrine pancreas positioning and head sizes are available at Mendeley under doi:10.17632/j78ddshctz.1

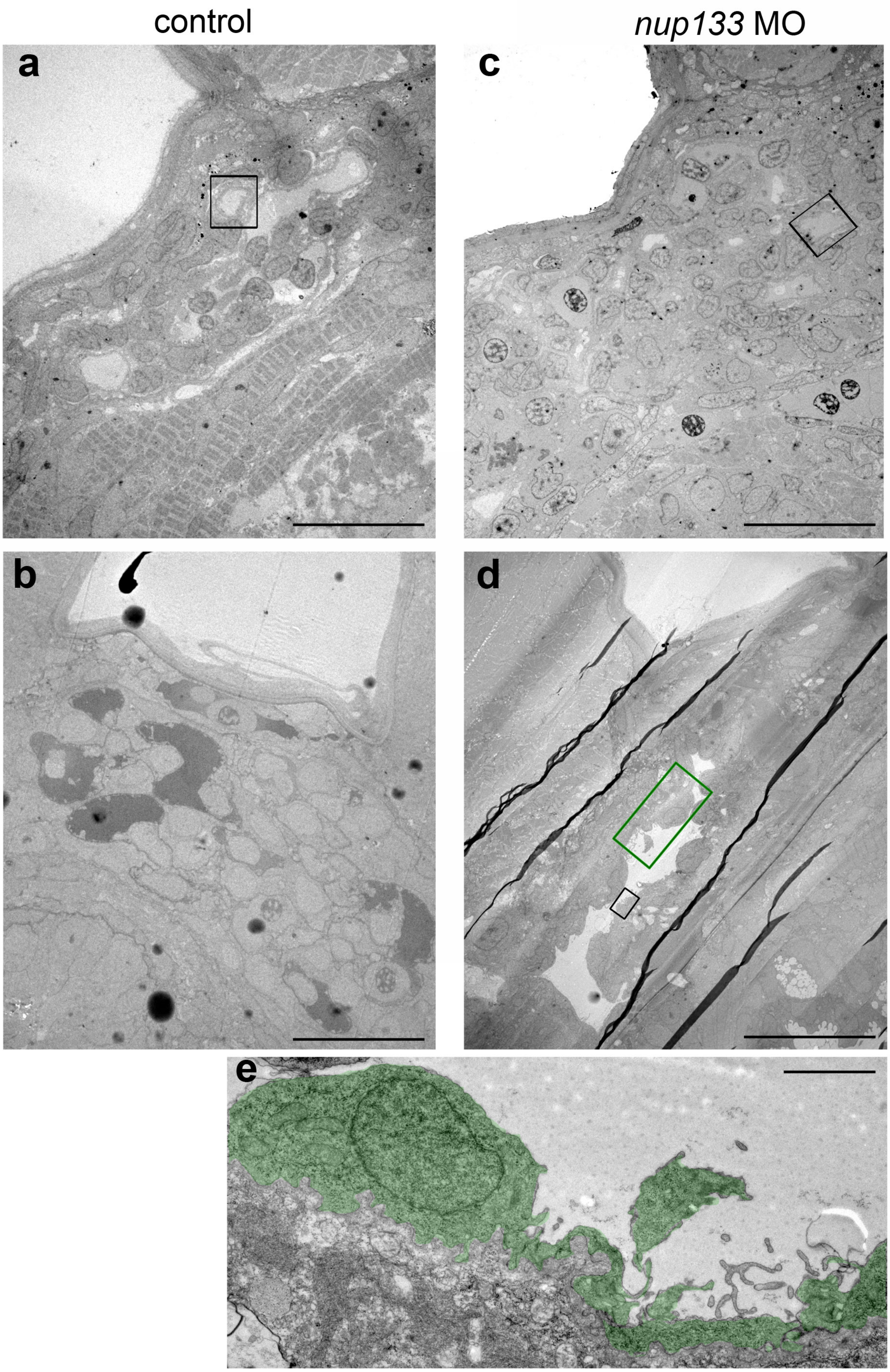


**Supplemental Figure S6. Transmission electron micrographs showing the glomerular area of 5 dpf uninjected control (a, b) and *nup133* MO injected larvae (c, d).** Higher resolution of the area indicated by the green rectangle in (d) is presented in (**e**). The black rectangles in (a), (c) and (d) correspond to areas presented at higher resolution in Figure 7 (a), (d) and (h), respectively, Scale bars 20 $\mu$m (a-d) and 2$\mu$m (e).

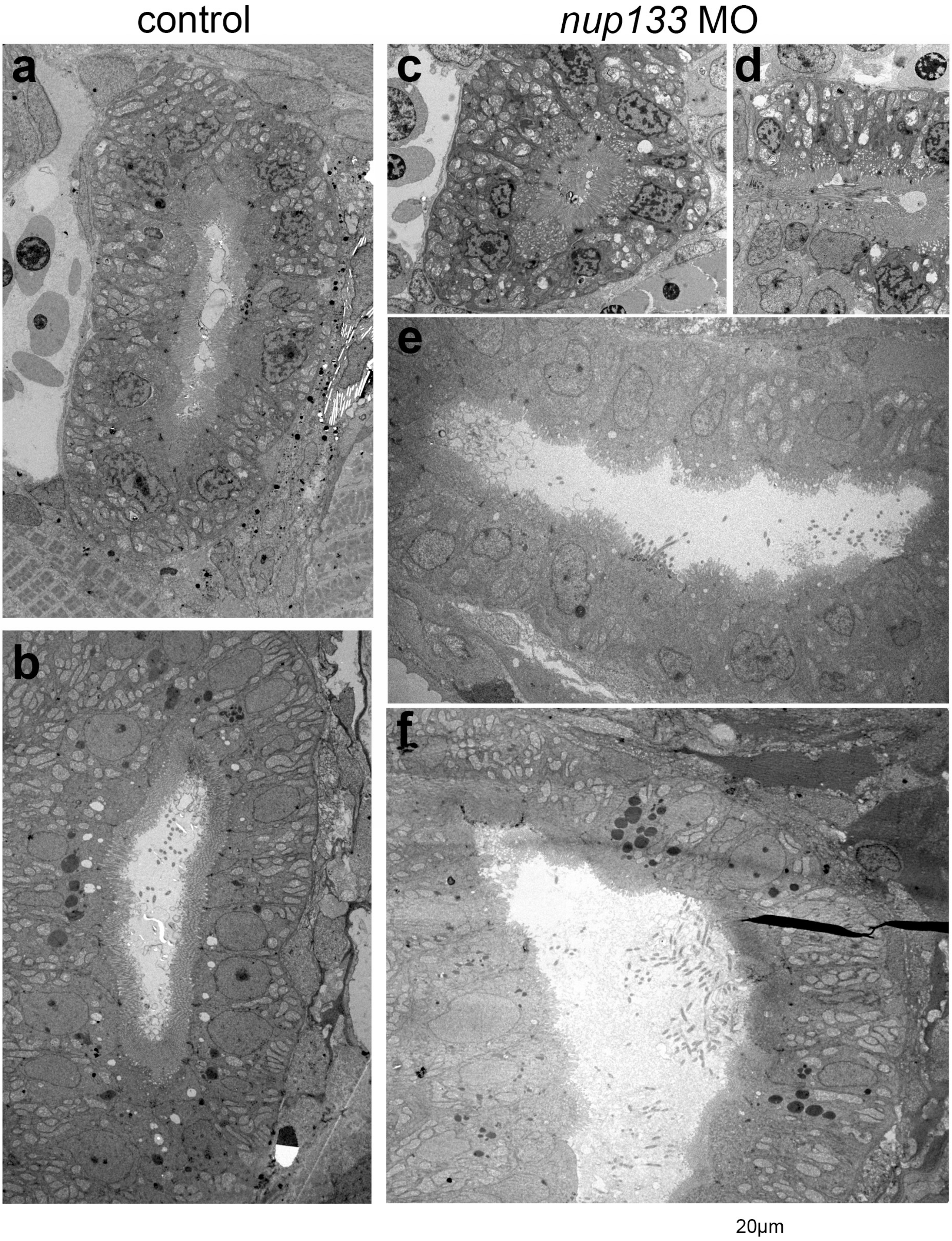


**Supplemental Figure S7. Transmission electron micrographs showing sections of proximal tubules from 5 dpf uninjected control (a,b) and *nup133* MO injected larvae (c, d, e, f).** Proximal tubules presented in panels (a), (c-d), and (f) arise from the same sections as the glomerular area presented in Supplemental Figure S6 (a), (c) and (d), respectively. Scale bar 20 $\mu$m.

**Supplementary Table S1: Oligonucleotides used in this study**

| | gene symbol | primer name | sequence (5' to 3') |
|---|---|---|---|
| **Cloning primers for ISH probes** | *nup133* | nup133-ISH-F | CGCAGTTCTATTATGACGCG |
| | | nup133-ISH-R | GCAGGTGACCACGGTACGAG |
| | *pax2a* | pax2a-ISH-F | AGACCCCTACCTGACGTG |
| | | pax2a-ISH-R | AGTCCAGGGTTCAGTGCT |
| | *cdh17* | cdh17-ISH-F | ACAGCTGGAGACCCTCAGAA |
| | | cdh17-ISH-R | GTCCTGAAGGCAGATGAAGC |
| | *wt1a* | wt1a-ISH-F | TGGCTGTCACACTCCTTCTG |
| | | wt1a-ISH-R | TAGGGTTTCTCCCCTGTGTG |
| **Morpholino sequences** | control | control MO | CCTCTTACCTCAGTTACAATTTATA |
| | *nup133* | nup133_E3I3 | GTCTCTTTTCTGACCTTCGCCACAG |
| | | nup133_I3E4 | CGAACAGCTGAACGGCAAAATAAAAC |
| **RT-PCR primers** | *nup133* | nup133-RT-F | CCAGACTTTCGGATCATCTC |
| | | nup133-RT-R | CGCTCAGGTCCAGAGAGATC |
| **RT-qPCR primers** | *nup133* | AL255-nup133_E3F | ATGGTTTGTGGAGAACGGCT |
| | | AL256-nup133_E4/5R | ACACTGATGGACTGAATGGGAG |
| | | AL259-Nup133_E3/4F | TCTGTGGCGAAGCTGTCGGT |
| | | AL260-nup133_E4/5R' | ATGGACTGAATGGGAGCCAGAT |
| | | AL239-nup133_E1/2F | CAGCACGATCTACTCCCACG |
| | | AL240-nup133_E2R | CCATCCAAGCCCAACCAGAT |
| | | AL249-nup133-E7/8F | CTGACGCCATCTGGGATTCA |
| | | AL250nup133-E9R | ATGCTTTCTGCAAGCGTGAC |
| | *actb2* | AL243-actb2-F | CGAGCTGTCTTCCCATCCA |
| | | AL244-actb2-R | TCACCACGTAGCTGTCTTTCTG |

## References to Supplemental Figures


1 Berke, I. C., Boehmer, T., Blobel, G. & Schwartz, T. U. Structural and functional analysis of Nup133 domains reveals modular building blocks of the nuclear pore complex. *J Cell Biol* **167**, 591-597, https://doi.org/10.1083/jcb.200408109 (2004).

2 Whittle, J. R. & Schwartz, T. U. Architectural nucleoporins Nup157/170 and Nup133 are structurally related and descend from a second ancestral element. *J Biol Chem* **284**, 28442-28452, https://doi.org/10.1074/jbc.M109.023580 (2009).

3 Berto, A. *et al.* Disentangling the molecular determinants for Cenp-F localization to nuclear pores and kinetochores. *EMBO Rep* **19**, https://doi.org/10.15252/embr.201744742 (2018).

4 Drin, G. *et al.* A general amphipathic alpha-helical motif for sensing membrane curvature. *Nat Struct Mol Biol* **14**, 138-146, https://doi.org/10.1038/nsmb1194 (2007).
